# Browning of freshwaters: Consequences to ecosystem services, underlying drivers, and potential mitigation measures

**DOI:** 10.1007/s13280-019-01227-5

**Published:** 2019-07-31

**Authors:** Emma S. Kritzberg, Eliza Maher Hasselquist, Martin Škerlep, Stefan Löfgren, Olle Olsson, Johanna Stadmark, Salar Valinia, Lars-Anders Hansson, Hjalmar Laudon

**Affiliations:** 1 Biology Department, Lund University, Ecology Building, Sölvegatan 37, 223 62 Lund, Sweden; 2 Department of Forest Ecology and Management, Swedish University of Agricultural Sciences (SLU), Skogsmarksgränd, 901 83 Umeå, Sweden; 3 Department of Aquatic Sciences and Assessment, Swedish University of Agricultural Sciences (SLU), P.O. Box 7050, 750 07 Uppsala, Sweden; 4 Stockholm Environment Institute, Linnégatan 87D, P.O. Box 242 18, 104 51 Stockholm, Sweden; 5 IVL Svenska Miljöinstitutet, Box 530 21, 400 14 Göteborg, Sweden; 6 Naturvårdsverket, 106 48 Stockholm, Sweden

**Keywords:** Acid deposition, Browning, Climate change, DOC, Iron, Land use

## Abstract

Browning of surface waters, as a result of increasing dissolved organic carbon and iron concentrations, is a widespread phenomenon with implications to the structure and function of aquatic ecosystems. In this article, we provide an overview of the consequences of browning in relation to ecosystem services, outline what the underlying drivers and mechanisms of browning are, and specifically focus on exploring potential mitigation measures to locally counteract browning. These topical concepts are discussed with a focus on Scandinavia, but are of relevance also to other regions. Browning is of environmental concern as it leads to, e.g., increasing costs and risks for drinking water production, and reduced fish production in lakes by limiting light penetration. While climate change, recovery from acidification, and land-use change are all likely factors contributing to the observed browning, managing the land use in the hydrologically connected parts of the landscape may be the most feasible way to counteract browning of natural waters.

## Introduction

**Fig. 1:**
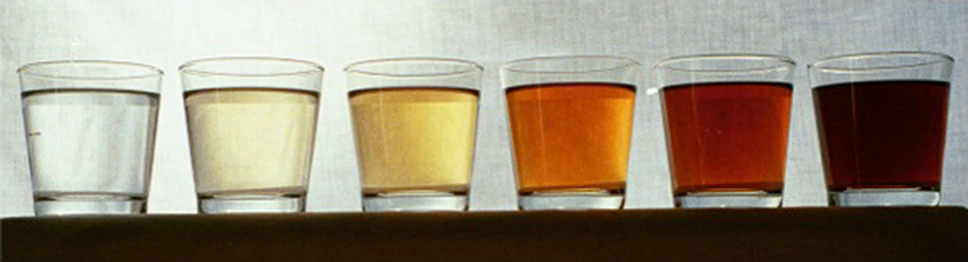
Differences in water color between surface waters due to differences in the concentration and composition of organic matter as well as iron. The water in the glasses comes from natural waters within a distance of 35 km in the County of Jönköping, southern Sweden. Photograph by Stefan Löfgren

**Fig. 2:**
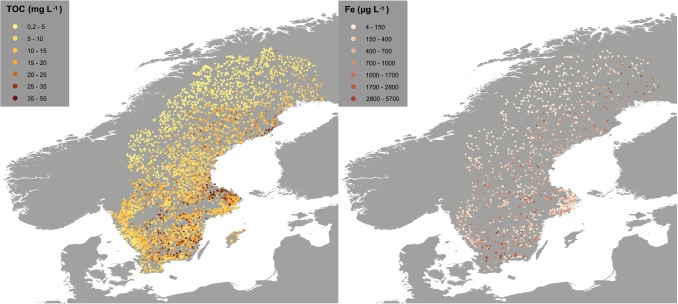
Concentrations of total organic carbon (left) and iron (right) in Sweden. The regional pattern is related to climate, soil type, and vegetation. Local variability is largely due to differences in water retention time. *Data source* The National Swedish Monitoring Program for surface waters carried out at by the Swedish University of Agricultural Sciences

The term browning (or brownification) refers to a considerable increase in water color (Fig. 1) reported for a vast number of northern freshwaters during recent decades (Monteith et al. 2007; de Wit et al. 2016). This browning has been mainly attributed to rising concentrations of terrestrially derived dissolved organic carbon (DOC), but rising iron (Fe) concentrations also contribute to the increase in water color (Weyhenmeyer et al. 2014; Fig. 2). Browning is an environmental concern in many freshwaters, since it has important ecological consequences by affecting water quality as well as the structure and function of the aquatic ecosystems (Solomon et al. 2015; Creed et al. 2018). Previous studies have suggested important implications also for ecosystem services that freshwaters provide, e.g., by affecting the fish community and production, and other aspects linking to recreational values (Van Dorst et al. 2018). Moreover, many freshwaters that are sources of drinking water are undergoing browning, with associated health risks and enhanced costs for production of clean and safe potable water (Lavonen et al. 2013).

While browning is observed on a vast geographical scale, not all waters are subject to this change. For instance, significant increases in DOC and Fe were found in 59 and 28% of investigated sites, respectively, in a study covering 340 surface waters in northern Europe and North America (Björnerås et al. 2017). Some of the largest and most dramatic increases have been recorded in boreal regions, which is likely due to the high amounts of organic carbon available for transport to water bodies. The boreal region is characterized by enormous stores of peat, containing at least one-third of the world’s terrestrial carbon pool (Bradshaw and Warkentin 2015). This large pool of soil carbon is several orders of magnitude more prone to degradation from climate warming than in other biomes (Crowther et al. 2016). Increases in DOC and Fe concentrations are also more frequent in freshwaters where coniferous tree covers dominate than in areas where, e.g., agriculture is the major land use (Björnerås et al. 2017), reflecting the relationship between soil pools and export of DOC and Fe to freshwaters (Weyhenmeyer et al. 2012). Furthermore, hydrological connectivity also regulates how DOC is transported from organic soils to streams (Laudon et al. 2011), suggesting that local and regional differences in climate and land use can enhance or reduce the effect on freshwaters. Finally, waters with a longer residence time, allowing for in-water processing and loss of DOC and Fe from the water column (Weyhenmeyer et al. 2014; Tiwari et al. 2017), are generally less responsive to browning.

There are still many uncertainties regarding the actual drivers and mechanisms behind browning, and consequently also pertaining to any measures that may be implemented to mitigate the continuing trend. It has been argued that the observed trajectory is driven by processes associated to *climate change*, which enhance terrestrial productivity, alter vegetation cover, and affect the hydrological control on production and transport of terrestrial DOC (de Wit et al. 2016; Finstad et al. 2016). Another proposed driver is the long-term change in *atmospheric sulfur* (*S*) *deposition*—which has declined strongly since the peak in the 1980s—allowing for recovery from acidification and increasing the solubility and transport of DOC from soils (Monteith et al. 2007; de Wit et al. 2016). Finally, a few recent studies invoke that *land*-*use change* may have a more important role to browning of freshwaters than previously thought (Schelker et al. 2012; Meyer-Jacob et al. 2015; Kritzberg 2017).

Sediment records indicate that DOC concentrations were significantly higher before the widespread human catchment disturbance, including forest grazing, farming, and land drainage, which intensified around ad 1500, and air pollution effects from around 1900, and these studies identify a DOC minimum around the early or mid-twentieth century (Meyer-Jacob et al. 2015). Regardless if current browning represents a return to what may be viewed as a more “natural state,” it has important negative consequences at several different levels. Thus, the motivation for this review is not to identify or strive toward a reference state with regards to water color, but to explore options to counteract browning. If land use and management are indeed factors involved, opportunities arise for local actions in order to mitigate browning.

Browning has been subject to considerable interest, and several scientific reviews effectively address its ecological effects and various aspects thereof (Solomon et al. 2015; Creed et al. 2018). Here, we aim at providing an interface between scientific knowledge and societal demands, i.e., to provide stakeholders and decision makers with potential measures to counteract browning, and thereby improve the ecosystem services provided by freshwater ecosystems. To this effect, we have organized the paper to provide an overview of consequences of browning for ecosystem services (Why is it a problem?) and of the underlying drivers and mechanisms (Where does the problem come from?), and with the emphasis on a discussion of possible mitigation measures (Are there ways to solve the problem?). We also address broader implications in terms of synergies and conflicts with other environmental issues. The options to mitigate browning should be viewed as hypotheses based on our current knowledge about controls of production and export of DOC and Fe in the landscape. In our final remarks, we provide specific suggestions for further research efforts and policy recommendations. Consequences, drivers, and mitigation measures of browning are vast areas to summarize, and therefore only a selection of relevant articles are cited, with priority laid to studies that are central and of relevance to environmental conditions of Sweden.

While browning is a problem in many regions, the initiative for this paper came from stakeholders in Sweden with interests in systems where browning has a tangible negative effect on ecosystem services, such as drinking water production, biodiversity, and recreational values. Therefore, we use Sweden as a starting point for our discussion, but most conclusions and suggestions apply to a wide range of lakes, streams, and rivers in boreal regions, and to browning as a general concern.

## Consequences of browning for ecosystem services

Numerous studies reveal strong impacts of browning on the organism structure and ecological processes in freshwaters. The concept of ecosystem services links ecological processes with human well-being, and is used here to illustrate which human benefits may be reduced or lost as browning proceeds. The consequences of browning are discussed following the four categories of ecosystem services—provisionary (drinking water production), cultural (outdoor experience), supportive (biodiversity), and regulating (biogeochemical processes). Moreover, browning may also have synergistic effects with other environmental threats, such as warming and eutrophication.

### Drinking water production

**Fig. 3:**
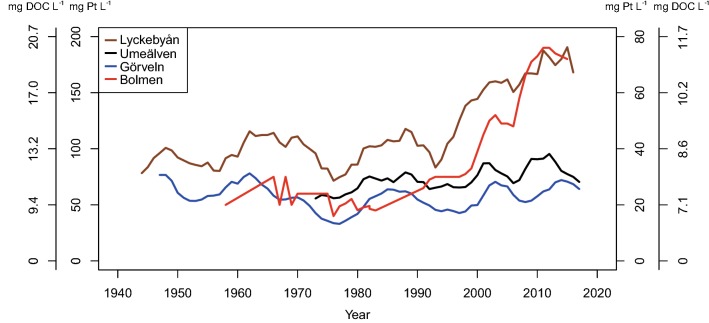
Long-term variability in water color and DOC in surface waters used for drinking water production (River Lyckeby (brown, left axis), Bolmen (red, right axis), River Ume (black, right axis), Görveln (blue, right axis). DOC is approximate, calculated from the relationship between measured water color and DOC in River Lyckeby. *Data sources* Municipality of Karlskrona (River Lyckeby), Kritzberg (2017) (Lake Bolmen), Swedish University of Agricultural Sciences (River Ume), and Norrvatten (Lake Görveln)

Clean water is one of the 16 UN sustainably development goals and plays a fundamental role for the development of societies, industrial and economic growth, as well as for human health. This imposes a strong incentive for society to provide drinking water of high quality. In many countries, surface waters (lakes and rivers) are the main source for drinking water production. In Sweden, for instance, 50% of the drinking water produced is drawn from surface waters, 25% from artificial infiltration of surface water, and 25% from groundwater (http://www.svensktvatten.se), suggesting that 75% of the drinking water supply is potentially influenced by browning (Fig. 3). In addition, lowering of groundwater levels, which is widely observed as a result of climate change and human outtake, will further increase our reliance on surface waters for drinking water production.

In drinking water-treatment plants (DWTPs), steps are included to reduce DOC concentrations. There are no legislative limits regarding DOC in drinking water, but there are recommendations, e.g. in Sweden, it is recommended that DOC concentrations stay below 4 mg L^−1^ (Köhler et al. 2016). Commonly, DOC is reduced in DWTP by chemical precipitation with FeCl_3_, AlCl_3_, or Al_2_(SO_4_)_3_. With browning, DOC concentrations in the incoming water increases, and more chemicals are required for precipitation, which is in direct conflict with general international legislation and recommendations to reduce chemical usage. The enhanced use of chemicals due to browning is estimated to increase the cost of producing drinking water by approximately 5% in Sweden. Moreover, precipitation of DOC with chemicals is never complete, and the remaining DOC interferes with subsequent purification steps, such as active carbon filters, UV-disinfection, and chlorination. Therefore, browning is also associated with risks that the drinking water contains elevated levels of pharmaceutical residues and potentially carcinogenic chlorinated organic compounds (Lavonen et al. 2013), and that microbial growth in the distribution network is enhanced. In addition to increasing concentrations of DOC, browning also comes with a change in the chemical character of DOC, and the chemical fractions differ in their amenability to removal and formation of disinfection byproducts (Ritson et al. 2014). Finally, if DOC concentrations are high (> 15 mg DOC L^−1^), chemical precipitation is no longer efficient, forcing DWTPs either to change their raw water source, which comes with major infrastructural costs, or to invest in ultra- or nanofiltration techniques.

### Outdoor experience

#### Brown water is seen as a negative factor for recreational value

There are general concerns about the impact of browning on recreational use and tourism (Valinia et al. 2012). Several studies have identified human preferences for clear water, for example, on Fraser Island in Australia, where only 1% of the respondents preferred to swim in brown lakes, whereas most people preferred clear lakes, streams, swimming pools, or the ocean (Hadwen and Arthington 2003). Moreover, in a study using geotagged photographs from social media, clear water increased the perceived recreational value of lakes, and visitors were willing to travel 56 min extra for every meter increase in water clarity (Keeler et al. 2015). Browning is also sometimes associated with increased abundances of the algae *Gonyostomum semen* (Rengefors et al. 2012), which can cause skin irritation and allergic reaction, thereby strongly reducing the recreational value.

#### Effects on fish and angling opportunities

At moderate levels, browning can promote fish production since terrestrially derived organic matter may act as an energy and nutrient subsidy (Finstad et al. 2014), and also reduce harmful UV radiation from the sun (Williamson et al. 1996). However, when the visibility for predatory fish decreases beyond a threshold level because of browning, their growth is impaired (Ranåker et al. 2014), rendering brown-water lakes less attractive for anglers. Moreover, primary production declines at high water color leading to lower overall food web production, including fish biomass (Karlsson et al. 2009). Algae produce unsaturated fatty acids essential for birds and mammals, and because browning often promotes phytoplankton species that produce less fatty acids, fish in brown lakes are less “nutritious” than fish in other lakes (Taipale et al. 2016). Humic substances, which dominate terrestrially derived DOC, also exert a mild chemical stress on many aquatic organisms (Steinberg et al. 2006), and have a complex role by both lowering pH and protecting against aluminum toxicity in fish (Serrano et al. 2008). Hence, the relation between water color and fish biomass is often described as hump shaped, where a mild browning increases fish production, whereas highly brown water reduces fish production (Finstad et al. 2014), and thereby angling opportunities.

### Biodiversity

Brown water and high DOC concentrations are generally associated with low productivity and diversity of phytoplankton communities (Jones, 1992), which often become dominated by mixotrophic species, i.e., algae that rely both on photosynthesis and heterotrophic uptake of bacteria or other algae (Wilken et al. 2018). One such species is *G. semen*, which often dominates in brown-water lakes (Trigal et al. 2011). However, although browning may have some effect on the phytoplankton species composition, it has a relatively limited effect on the diversity (Lebret et al. 2018). Moreover, a direct effect of the reduced light penetration is that pelagic species become favored over benthic species (Ask et al. 2009), and that macrophyte species with capacity to elongate their stem and reach the surface, such as *Elodea canadensis*, may become dominant (Mormul et al. 2012).

### Biogeochemical consequences that follow with browning

In the last decade, freshwater DOC has been recognized as a major component of the global carbon cycle, and inland waters have been identified as key contributors to C emissions (as a greenhouse gas) to the atmosphere (Tranvik et al. 2009). The terrestrial DOC that is exported to surface waters is to a large extent processed there, either mineralized to carbon dioxide or methane that is emitted to the atmosphere, or aggregated and sedimented (Tranvik et al. 2009). Browning and rising DOC concentrations could therefore increase the flux of greenhouse gases to the atmosphere.

Since both DOC and Fe act as carriers of other elements, changes in many chemical variables occur as a consequence of browning. For instance, DOC influences the speciation, mobility, and bioavailability of most cationic metals including mercury (Hg; Lydersen et al. 2002). Higher Hg levels in fish and invertebrates are generally found in aquatic systems with higher DOC concentration (Creed et al. 2018). Aluminum, zinc, and copper have increased widely, partly linked to the increased browning as DOC forms strong organic complexes, which favor mobilization and transport from soils to surface waters (Oni et al. 2013). Iron also acts as an important vector of highly toxic metals such as lead, arsenic, and vanadium, which have increased in concentrations in many waters (Wällstedt et al. 2010). Browning also brings micro- and macronutrients that can support phytoplankton production, although it is only at rather low DOC concentrations that this fueling effect on primary production overrides that of increased light attenuation (Creed et al. 2018). Finally, browning affects the recovery from acidification as DOC acts as an organic acid and hence reduces the expected recovery associated with the decline in mineral acid deposition (Futter et al. 2014).

### Synergies with other aspects of environmental change

Browning interacts with other large-scale environmental changes, such as climate warming and eutrophication, which may lead to additive or even synergistic effects. For example, eutrophication and browning will together reduce water clarity more than each of them alone and thereby reduce the area available for benthic macrophytes, and the feeding success of predatory fish (Ranåker et al. 2014). Although browning alone may not always stimulate cyanobacteria (Hansson et al. 2013), the cyanobacteria *Microcystis* spp. benefit when climate warming and browning act together (Urrutia-Cordero et al. 2016). Most studies on browning have been performed in freshwater systems, but large amounts of humic substances also enter the sea through rivers (Svedäng et al. 2018). Although browning effects in saltwater are still less pronounced than in freshwaters, phytoplankton production is suppressed, thereby shifting the carbon flow toward microbial heterotrophy also in coastal areas of the sea (Wikner and Andersson 2012). In coastal regions where climate warming will lead to higher precipitation, and thereby higher runoff, this may eventually result in reduced fish production.

## Drivers and mechanisms

**Fig. 4:**
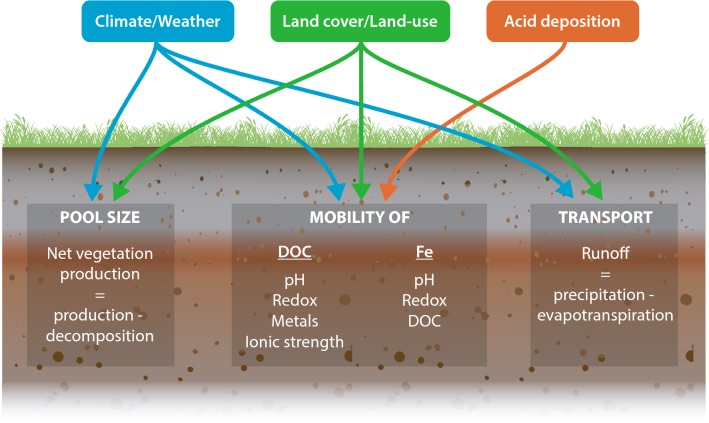
DOC and Fe are mobilized and exported from the catchment. The amount of organic carbon that exists in surrounding soils sets a first constraint on how much can potentially be transported to surface waters. The pool size of organic carbon is the net production of terrestrial vegetation. The source of Fe, on the other hand, is the bedrock and soil minerals. Only a minor fraction of that organic carbon and Fe will be available for transport, so a second constraint is the mobility of DOC and Fe in soils. For DOC, this is controlled by factors such as pH, redox conditions, cation adsorption, and ionic strength. Fe mobility is controlled by pH, redox, and DOC concentration. The third constraint is the actual transport driven by runoff, which is the difference between precipitation and evapotranspiration. Studies addressing underlying drivers of browning generally focus on three groups of drivers: climate/weather conditions, land cover/land use, and acid deposition. The way these drivers affect pool size, mobility, and transport of organic matter is elaborated in Box 1

The principal drivers that have been proposed to contribute to the widespread browning are climate change/weather patterns, acid deposition, and land cover/land use. The mechanisms by which these different drivers may affect export of DOC and Fe from surrounding soils are outlined in Fig. 4 and Box 1. To the extent that reduced acid deposition has been the major driver, browning will likely eventually level off and represent a return to a more “natural state” (Monteith et al. 2007; de Wit et al. 2016). If reduced acid deposition were the sole driver of browning, we would stand without measures to counteract it. However, some historical records do not corroborate that water color was suppressed in response to increasing S deposition (Kritzberg 2017), pointing to the role of other drivers than just recovery from acidification to browning. Other studies suggest that increasing primary production and accumulation of soil organic carbon, driven primarily by expanding intensive coniferous forestry and amplified by longer growing seasons and varying precipitation patterns, are major factors behind the current browning trend (Finstad et al. 2016).

Time series analyses clearly demonstrate that oscillations in water color with rather large amplitude relate to weather/climate variability, e.g., dry versus wet periods (Erlandsson et al. 2008). While browning on the centennial scale thus far does not seem to be primarily explained by any long-term trend in precipitation (Kritzberg 2017), a further increase in precipitation will likely enhance browning, except for systems in high precipitation areas (de Wit et al. 2016).

Thus, what we can say with certainty, based on current knowledge about DOC and Fe mobilization, is that water color will continue to vary widely in response to variable weather and air pollution loads, and is likely to continue to pose environmental and aesthetic problems. Acknowledging the human influence on terrestrial primary production and organic carbon accumulation means that our best chance to control/mitigate browning is by managing the land use in the hydrologically connected parts of the landscape (Fig. 4).

**Box 1:** Drivers and mechanisms that affect DOC and Fe concentrations

**Atmospheric acid deposition** Sulfate (${\text{SO}}_{4}^{2 - }$) deposition has varied tremendously over the last century, with a sharp increase after the Second World War, followed by a sharp decline as a result of international legislation. Since the peak deposition in the early 1980s, ${\text{SO}}_{4}^{2 - }$ concentrations in soils have decreased drastically (Akselsson et al. 2013). The low pH and high ionic strength, which follow with high S deposition, may reduce solubility and mobility of DOC in soils. Field experiments show that the chemistry of current-day precipitation promotes higher DOC concentrations in organic-rich soil horizons of podzols (Ekström et al. 2011). In contrast, the dominant trend since the late 1980s is declining DOC concentrations in soil solution at 50 cm soil depth (less organic rich, Löfgren and Zetterberg 2011). In the boreal and nemoboreal landscape, organic-rich soils in riparian zones and peatlands with high connectivity to the streams are the most important DOC sources (Laudon et al. 2011). This probably explains why long-term monitoring shows that increases in DOC concentrations in freshwaters correspond with declines in ${\text{SO}}_{4}^{2 - }$ concentrations (Monteith et al. 2007; Erlandsson et al. 2008), while soil solution in less organic-rich soil horizons shows the opposite trend. However, since monitoring generally started during peak deposition, the freshwater studies cannot resolve whether current browning is a return to a more natural, pre-acidification state. The few studies, where historical data from before peak acidification are included, report no decline in water color in response to the increasing S deposition, suggesting that S deposition may not always be the primary factor behind browning on a multidecadal scale (Johansson et al. 2010; Kritzberg 2017). Iron export is also potentially influenced by S deposition, although the relationship is more complex. While solubility of Fe decreases with the increasing pH, solubility of DOC, which is critical for Fe mobility, is positively affected (Ekström et al. 2011). Fe mobility could also be affected directly by the availability of S, e.g., by precipitation of FeS under strongly reducing conditions. However, studies that address the effects of S deposition on Fe export are sparse, and the results are inconclusive (Björnerås et al. 2017).
**Climate drivers** Temperature and precipitation have increased in Fennoscandia during recent decades (Nikulin et al. 2016). Precipitation promotes DOC export to surface waters only by increasing the water runoff through soils, but also by raising the water table and increasing connectivity between organic soils and surface waters (Laudon et al. 2011). High precipitation and temperature further enhance microbial decomposition and promote reductive dissolution of precipitated Fe(III) hydroxides to the more mobile Fe(II), and release the adsorbed DOC (Knorr 2013). Moreover, faster flushing rates, driven by increasing precipitation, may restrain Fe sedimentation and DOC processing and thus result in elevated Fe and DOC concentrations in surface waters (Weyhenmeyer et al. 2014; Tiwari et al. 2017). Accumulation of DOC and increasing soil acidity during drought events and subsequent flushing have also been shown to increase DOC export, but the long-term effect of rewetting events on DOC export is modest (Clark et al. 2012). While the amount and intensity of precipitation are good predictors for inter-annual variability in water color, it is less clear how important they are for long-term trends (Erlandsson et al. 2008). Finally, longer growing seasons due to the increasing temperatures, have been related to increased terrestrial organic carbon production, decomposition, and export to surface waters (Finstad et al. 2016).
**Land use** Land cover has long been recognized as the best predictor of water color in freshwaters, and DOC and Fe concentrations are positively related to the proportion of wetland and coniferous forest in the catchment (Mattsson et al. 2009). Paleolimnological studies clearly demonstrate that landscape utilization has strongly influenced lake DOC at the centennial scale (Meyer-Jacob et al. 2015). During the last century, land cover in many densely populated areas has gone from an open landscape with low-intensity agriculture to a dominance of coniferous forestry (Lindbladh et al. 2014). Compared to agricultural and deciduous forest land cover, coniferous forest soils have higher DOC and Fe leaching potential (Li and Richter 2012; Camino-Serrano et al. 2014). Due to the slow buildup of soil organic carbon pools, the effect of afforestation on water color is likely to occur gradually, with a lag of several decades (Kritzberg 2017). Long-term, historical data show that afforestation may play a major role in explaining browning in southern Sweden (Kritzberg 2017). Other aspects of land management also influence DOC and Fe export. Clear-cutting and forestry soil preparation have been shown to increase short-term DOC leaching, but it is unclear how long-term export is affected (Schelker et al. 2012). Groundwater manipulation by ditching also affects DOC and Fe leaching, but the effects vary with the soil type as is elaborated in the “Mitigation measures” section.

## Mitigation measures

### Background

The DOC flux to headwater streams is largely determined by the quantity and quality of organic matter in wetlands (Creed et al. 2008) and organic-rich riparian forest soils (McGlynn and McDonnell 2003), which provide the main sources for downstream environments (Tiwari et al. 2017). Thus, to properly understand the spatial and temporal aspects of DOC in surface waters hydrological connectivity and transport mechanisms between soil organic matter (SOM) sources and surface waters must be considered simultaneously (Laudon et al. 2011). The quantity and quality of the exported DOC depend largely on land use that dictates the type of vegetation cover (Kothawala et al. 2015; Kritzberg 2017). How and when SOM pools are linked to surface waters depends on the groundwater level and subsurface groundwater flow paths (Laudon et al. 2011). When the groundwater level is high and the organic soil layers saturated, water is in close contact with relatively recently deposited organic matter within the surface peat that leaches from the SOM pool (Nieminen et al. 2018). During precipitation events, or snowmelt, groundwater levels peak and DOC export to surface waters is at its highest (Laudon et al. 2011). Generally, subsurface groundwater flow paths follow surface topography and are concentrated in localized areas in the glacial till landscape, which feed into streams at discrete points (Ploum et al. 2018), making them important controls for water quality. Once in the surface water, the retention time affects the time during which DOC can be processed and lost (Stanley et al. 2012).

Understanding the linkages between the distribution and quality of the SOM, groundwater level and flow paths, and in-water processing, can help us to identify ways in which they can be managed to counteract ongoing and future browning. Below we discuss a number of potential mitigation measures related to altered land use (i.e., vegetation type) in hydrologically connected areas, modifying hydrological connectivity in itself, and restoring retention time, all as measures to reduce browning of waters.

### Using hydrological connectivity to target changes in land-use management

The importance of the riparian zone, both in terms of providing a major source of DOC and controlling and buffering hydrological connectivity and DOC fluxes from surrounding hillslopes, has been highlighted in various environments (McGlynn and McDonnell 2003; Marwick et al. 2014; Musolff et al. 2018). This is especially true in boreal regions where the riparian zone has a critical role in DOC export because of high accumulation of SOM (Dick et al. 2015). Boreal soils tend to generate significantly higher DOC concentrations than other forested biomes because of high conifer cover with poor litter quality and relatively wet and cold soils with slow decomposition. The increased cover of Norway spruce (sometimes termed “sprucification”) that has occurred since pre-industrial times due to forest industry demands and modern forest management practices (Hellberg et al. 2009), has important implications for DOC dynamics. In addition, suppression of natural disturbance regimes, such as recurrent wildfires in upland forests (Hellberg et al. 2009) and seasonal floods in riparian environments (e.g., Johansson and Nilsson 2002), have favored the species composition toward conifers. This shift from deciduous to conifer tree dominance is particularly important for riparian areas and may therefore be an important player in the browning of surface waters.

**Fig. 5:**
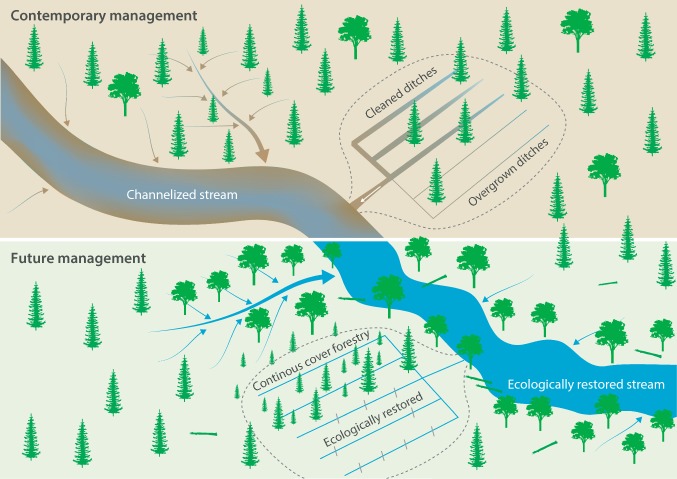
Contemporary (upper panel) versus future management states (lower panel) of riparian zones, distinct riparian inflow points (DRIPS), streams, and drained peatlands. Arrows show groundwater flow direction toward a stream, with most of the water entering the stream at DRIPS. Size of the arrows indicates the amount of groundwater flow, and their color indicates the relative amount of DOC in that water (brown = high DOC while blue = less DOC). In the contemporary state, spruce or pine dominates, whereas in the future management state, deciduous trees have been restored in DRIPS and riparian zones. Channelized stream reaches have been straightened and narrowed, and in-stream boulders moved to the sides of channels to make levees. Ecologically restored reaches have levees dismantled, and large boulders and in-stream wood added to the stream to increase hydrogeomorphic complexity and increase retention time. Drained peatlands show a few examples of contemporary and potential future management options for these areas; see Fig. 6 and supporting text for discussion of additional options for management of drained peatlands

However, not all riparian zones behave the same way, and are therefore not equally important for the DOC export. Recent work has illustrated this by quantifying the contribution of water from the riparian zone, showing that 70% of hydrologic inputs into a 1400 m stream segment enter at only a few distinct groundwater input zones, which constitute less than 10% of the channel length (Leach et al. 2017). These so-called discrete riparian input points (DRIPS, Ploum et al. 2018) are the result of the topography channelizing groundwater water into specific areas, acting as hotspots for water and solute input to streams (Fig. 5). As such, these highly organic sites are also likely to contribute with large amounts of DOC to surface waters and thus, are suitable targets for active management.

#### Riparian zones: Shift species composition to deciduous

The century-long transition toward dominance of conifers has likely resulted in increased DOC concentrations in many surface waters. In general, litter from coniferous species, such as spruce and pine, is a lower-quality substrate for microbial processes compared to deciduous trees (Duan et al. 2014), and more DOC leaches from coniferous forests than from deciduous (Camino-Serrano et al. 2014). Thus, the ongoing browning trend may be halted or even reversed by management of spruce via thinning and actively planting deciduous broadleaf tree species in riparian areas and DRIPS, thereby reducing the DOC entering freshwaters (Fig. 5). A higher density of deciduous streamside vegetation may, besides lowering stream DOC export, also promote headwater biodiversity (Jonsson et al. 2017). Hence, although management actions toward more deciduous riparian forest cover could potentially take decades (Hasselquist et al. 2015) or up to centuries to affect the DOC concentration in surface waters, such a transition would also have several other more instant positive ecosystem effects.

### Manipulating hydrological connectivity

Extensive peatland areas throughout Fennoscandia have been drained, in more or less systematic ways, with the purpose to improve forest production (Päivänen and Hånell 2012; Sikström and Hökkä 2016). Area drained for peatland forestry has been positively correlated with TOC and DOC (Marttila et al. 2018) and drained peatland forests have been shown to be significantly greater sources of DOC to receiving waters than undrained peatlands and upland forest soils (Rantakari et al. 2010). Päivänen and Hånell (2012) suggested that in drained peatlands, aeration promotes microbial processes and solubility, and thereby increases peat mineralization, resulting in the increasing DOC concentration. This increase in peat decomposition level due to drainage, combined with naturally fluctuating water tables, creates a condition with increased loads of DOC and nutrients from drained peatlands (Marttila et al. 2018; Nieminen et al. 2018).

#### Ditch network maintenance (DNM)

**Fig. 6:**
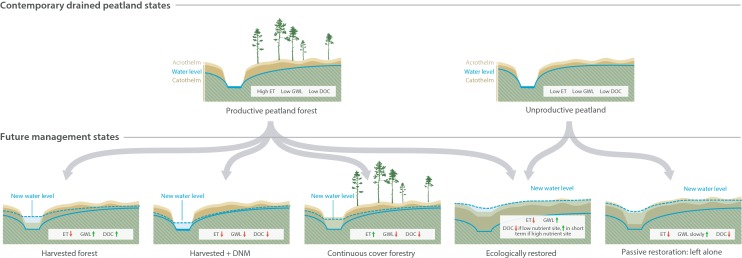
Flow chart showing how management actions on drained peatlands could affect DOC. When a productive peatland forest has reached maturity, there are a number of options for managing the ditches within these stands: (1) harvest of all trees which will reduce evapotranspiration (ET), increase ground water level (GWL), and increase DOC. (2) If ditch network maintenance (DNM) is paired with harvest, then GWL will likely lower and DOC will lower (but depends on interaction of GWL with what is left of the subsided/degraded peat). (3) With continuous cover forestry (and also late in-stand rotation), the high ET of the site keeps GWL and DOC low without applying DNM. (4) Productive peatland forests can also be ecologically restored by blocking ditches to reestablish the natural hydrology of the peatland and by cutting trees to decrease the ET, both of which increase the GWL, and for productive, high nutrient sites, can temporarily increase DOC during the first year after restoration. Unproductive drained peatlands have fewer management options, such as (1) ecological restoration—which does not affect DOC in this case or (2) passive restoration, by just being left without measures. Passive restoration happens over decades/centuries with natural ingrowth of sphagnum moss which blocks the ditches, slowly increases GWL, and increasing DOC to natural levels for peatlands. Water level is key to restoration success because ‘over-restoration’ can increase DOC for all types of sites

In Sweden, there is currently no new drainage ditch digging performed in pristine peatlands. However, DNM is done to restore the drainage functions of degraded ditches in order to improve and maintain the forest production achieved by the first-time drainage (Fig. 6; Sikström and Hökkä 2016). DNM can also involve digging of supplementary ditches in-between existing ones to increase the drainage function. DNM in Sweden is typically done at stand rotation to lower groundwater levels in order to allow for seedling establishment, since the removal of the mature tree stands reduces evapotranspiration (Sikström and Hökkä 2016). In Finland, it is more common that DNM is done every 20 years, typically in connection with thinning operations (Sikström and Hökkä 2016). It should be noted that environmentally responsible drainage becomes more difficult to implement with the increasing time after drainage because peat decomposition increases its erodibility as well as the peat subsidence (Päivänen and Hånell 2012).

How DNM affects water quality is a hot topic, and a recent review on the subject summarized the current understanding of the impact of DNM on water quality, including DOC concentrations (Nieminen et al. 2018). In general, the effect of DNM on DOC export depends on the underlying soil type. For peat covering fine-textured mineral soils or deep peat soil, a significant decrease in DOC concentrations after DNM has been found to continue for up to two decades, while no such effect has been observed for peat covering coarse-textured mineral soils (Nieminen et al. 2018). Nieminen et al. (2018) also summarized the proposed hypotheses for the processes likely causing decreased DOC exports from DNM areas. They argued that the most frequently suggested mechanism is that DNM changes the water pathways so that it no longer comes into close contact with high-quality, recently deposited organic matter within the surface peat (Fig. 6). Instead, water flows in deep soil, where it can interact with positively charged mineral soil oxyhydroxides that act as sorption sites for negatively charged organic moieties (Åstrom et al. 2001). If the water table is already low due to high evaporative demand from mature trees (Fig. 6; Sarkkola et al. 2010), negligible changes in DOC exports are to be expected (Nieminen et al. 2018). DNM may, however, induce other water-quality problems primarily related to erosion of suspended solids (Nieminen et al. 2018). Hence, remedial actions against browning must be valued against other negative water-quality impacts.

#### Forestry methods: Reduce disturbance and hydrological connectivity of production peatland forests

Even-aged forest management, which includes clear-cutting, site preparation to create planting spots, and sometimes DNM, has been shown to increase DOC export to streams significantly. In northern Sweden, clear-cutting increased downstream DOC export by 92%, and site preparation increased it by 195%, compared to pretreatment conditions (Schelker et al. 2012). The increase in DOC export is likely primarily due to the raised groundwater level following harvesting (Fig. 6; Schelker et al. 2012), which during high-flow conditions are caused by the lateral flow through riparian zones that exponentially increases DOC concentrations of riparian soil water near the soil surface. In Finland, where the typical forest regeneration phase in even-aged forest management includes clear-cutting, soil preparation for planting as well as management of the existing ditch networks, DOC exports increase, especially from the most fertile sites (Nieminen 2004; Kaila et al. 2016). If continuous cover forestry was implemented, the rise in groundwater level due to forest harvest would be reduced due to the evapotranspiration of the remaining tree stand (Fig. 6; Sarkkola et al. 2010). This would plausibly result in less flow through the most peat-rich riparian zone layers and hence reduce the mobilization and release of DOC, redox-sensitive nutrients and metals (Nieminen et al. 2018). Recent studies have indicated that it is a change in redox-conditions in surface peat that is the key factor controlling the enhanced DOC exports (Nieminen et al. 2018) from drained peatland forests after clear-cutting.

### Manipulating water residence time

Human impact has in many cases caused reduction of water residence time through channelization, construction of canals and levees, as well as elimination of wetlands or floodplains that would otherwise slow water movement (Stanley et al. 2012). Decreases in residence times are particularly pronounced in urban settings, where impervious surfaces result in rapid downstream routing of water by preventing infiltration into soils and groundwater (Stanley et al. 2012). Similar hydrologic short-circuiting also occurs in agricultural areas with tile drains and forest drainage ditches through peatlands described above.

Once terrestrial DOC is delivered to the aquatic environment, its quantity and quality can be modified by microbial processing, respiration, sedimentation, adsorption/desorption, photobleaching, and photooxidation (Stanley et al. 2012). A few active measures are available to increase the retention time of water in a system and thereby enhance these processes. Some key actions are to restore the long residence time associated with a hydrological regime more typical of natural peatlands or to increase geomorphic complexity of streams through re-meandering or restoring in-stream heterogeneity.

#### Restoring peatlands

Restoration of historically drained peatlands is often performed in order to improve water quality, but there are conflicting results on how restoration affects DOC export. Restoration is a disturbance in itself; ditches are blocked by damming and in-filling usually using peat material in order to raise the water table level back to the level prior to drainage (Fig. 6; Laine et al. 2011). In the first few years after restoration, DOC exports can sum up to several years’ worth of background export (Koskinen et al. 2017). This has primarily been the case in drained nutrient-rich peatlands where there is a significant risk for increased short-term export of DOC. In contrast, restoration of nutrient-poor peatlands has been shown to pose less risk (Koskinen et al. 2017). Over the long-term, the reduction of DOC may counterbalance this initial pulse, but how long this takes is still unclear. After 4 years, DOC had recovered to near natural conditions in one study (Koskinen et al. 2017), while in a study of 24 restored peatlands, restoration reduced DOC concentrations to near natural or reference levels in most mire types within 6 years (Menberu et al. 2017). Further support for reduced DOC from restored peatlands comes from a national survey in the UK suggesting that the water sampled from restored drainage ditches tended to be less colored than that from unblocked drains (Armstrong et al. 2010). Furthermore, the degree of restoration also affects DOC in peatlands. Hydrologically over-restored sites (inundated) have significantly higher concentrations of pore water DOC than less restored sites (Menberu et al. 2017), indicating the importance of achieving a hydrological regime typical of natural peatlands during restoration.

#### Stream restoration

Overall, streams and rivers are increasingly being recognized for their significant role in the processing of organic matter and DOC (Mineau et al. 2016). In a study of seven watersheds in the northeastern US, between 27 and 45% of the water column DOC was removed by in-stream processing during transport (Mineau et al. 2016). In contrast, other studies have shown that there is little or no processing of DOC relative to water residence time. For example, first to fourth order (< 0.5–4 m wide) boreal streams were found to act as passive pipes since in‐stream processing of DOM was restricted by short water residence times (Kothawala et al. 2015).

Theoretically, increasing residence time of water in the stream system through increasing hydrogeomorphic complexity should lead to reduction of DOC, but it is unclear to what extent restoration can affect this, and little empirical work has been done to test this in a restoration context. Changing the load or concentration of DOC in streams and rivers rarely motivates management activities—rather reduction in nutrients such as nitrogen and phosphorus are commonly stated goals (Stanley et al. 2012). However, restoration through addition of large boulders and in-stream wood has been shown to increase the retention of allochthonous organic matter (Frainer et al. 2018), as well as affect hydrodynamics and surface water–ground water interactions of streams (Kupilas et al. 2017). For example, stream widening reduces flow velocity and creates backwaters and pools which could change the size and location of transient storage zones, which in turn would enhance carbon processing and nutrients (Kupilas et al. 2017). Little work has been done in boreal and temperate contexts, but in the subtropical, low gradient, blackwater-type (high DOC) Kissimmee River in Florida, USA, primary production and respiration were estimated before and after canal backfilling and restoration of continuous flow in the natural stream channel (Colangelo 2007). After the restoration, net daily metabolism values indicated an increase in the amount of organic matter being processed, likely originating from the reconnected floodplain. DOC values decreased after restoration, but not significantly in the short 3-year period of postrestoration measurements (Colangelo 2007). As more restoration is planned at the watershed scale, more research is needed on how restoration of in-stream complexity affects ecosystem processes such as metabolism, and specifically, DOC processing.

## Final remarks

Changing climate/weather, reversed acidification, and altered land-use and management practices have acted in concert to generate the increased browning of inland waters observed over the last few decades. Although it is difficult to disentangle the individual importance of each of these drivers in space and time—making it difficult to predict future trajectories for water color—variability in climate drivers will continue to promote high concentrations of DOC and Fe at levels that pose a problem to stakeholders and impair ecosystem services. However, the growing recognition that land use and management are significant drivers provides us with the potential measures to mitigate or reduce browning. Unfortunately, as the role of land use and management has been largely overlooked, research has not been directed toward evaluating such measures.

Therefore, the options to mitigate browning, which are highlighted and discussed here, should be regarded as hypotheses based on the contemporary understanding of what factors govern the production and export of DOC in the boreal landscape and its loss in the aquatic system. Interestingly, several of the proposed interventions are being implemented at small scales, but for other purposes. Actively promoting deciduous broadleaf tree species in riparian areas, for instance, is becoming a more common practice, since there are known benefits for biodiversity and fishing opportunities. Similarly, restorations of peatlands and wetlands are performed both in the agricultural and forested landscape, with the aims to increase biodiversity and nutrient retention, as well as to reduce greenhouse gas emissions from decomposing peat. For an optimized management perspective, it is imperative that funding is allocated to evaluate the multiple consequences of such interventions, including DOC and Fe export. A dialog between land owners, forest companies, scientists, and policy makers should be promoted in order to improve and spread knowledge about how land-use management affects water quality and other ecosystem services, and to build commitment. Finally, funding to perform large-scale and long-term experiments, designed to test the efficiency and response over time is crucial for providing decision support regarding management practices. Some measures may be in conflict with other water-quality issues such as erosion of suspended solids, or other ecosystem services, such as forest production and associated carbon sequestration. However, if measures can be efficiently assessed and directed toward hydrologically connected areas only, such conflicts may potentially be overcome.
